# Function-Based Discovery of Significant Transcriptional Temporal Patterns in Insulin Stimulated Muscle Cells

**DOI:** 10.1371/journal.pone.0032391

**Published:** 2012-03-01

**Authors:** Barbara Di Camillo, Brian A. Irving, Jill Schimke, Tiziana Sanavia, Gianna Toffolo, Claudio Cobelli, K. Sreekumaran Nair

**Affiliations:** 1 Information Engineering Department, University of Padova, Padova, Italy; 2 Division of Endocrinology, Metabolism, Nutrition, and Internal Medicine, Mayo Clinic Rochester, Rochester, Minnesota, United Sates of America; Istituto Dermopatico dell'Immacolata, Italy

## Abstract

**Background:**

Insulin action on protein synthesis (translation of transcripts) and post-translational modifications, especially of those involving the reversible modifications such as phosphorylation of various signaling proteins, are extensively studied but insulin effect on transcription of genes, especially of transcriptional temporal patterns remains to be fully defined.

**Methodology/Principal Findings:**

To identify significant transcriptional temporal patterns we utilized primary differentiated rat skeletal muscle myotubes which were treated with insulin and samples were collected every 20 min for 8 hours. Pooled samples at every hour were analyzed by gene array approach to measure transcript levels. The patterns of transcript levels were analyzed based on a novel method that integrates selection, clustering, and functional annotation to find the main temporal patterns associated to functional groups of differentially expressed genes. 326 genes were found to be differentially expressed in response to *in vitro* insulin administration in skeletal muscle myotubes. Approximately 20% of the genes that were differentially expressed were identified as belonging to the insulin signaling pathway. Characteristic transcriptional temporal patterns include: (a) a slow and gradual decrease in gene expression, (b) a gradual increase in gene expression reaching a peak at about 5 hours and then reaching a plateau or an initial decrease and other different variable pattern of increase in gene expression over time.

**Conclusion/Significance:**

The new method allows identifying characteristic dynamic responses to insulin stimulus, common to a number of genes and associated to the same functional group. The results demonstrate that insulin treatment elicited different clusters of gene transcript profile supporting a temporal regulation of gene expression by insulin in skeletal muscle cells.

## Introduction

Skeletal muscle is responsible for about 65% of glucose disposal following a meal [1] and reduced insulin induced glucose disposal results in impaired glucose tolerance. *In vivo*, insulin plays an important role in the regulation of skeletal muscle glucose uptake and regulation of skeletal muscle protein, amino acid and fatty acid metabolism [2], [3]. Acutely, insulin stimulates glucose uptake in skeletal muscle cells by accelerating the recruitment of GLUT4 to the sarcolemma [4]–[7], a process that is exquisitely regulated by a specific insulin-responsive protein signaling cascade (i.e., IR/IRS1/PI3K/Akt) [4], [5], [8], [9]. Similarly, insulin acutely stimulates protein synthesis (in the presence of adequate amino acids) by also activating a specific insulin-responsive protein signaling cascade (i.e., Akt/mTOR/S6K) [10]–[14]. Both of these responses are regulated by reversible post-translational modifications (i.e., phosphorylation) of key signaling protein molecules. However, less information is available about insulin impact on gene transcription that also may affect insulin action: it is currently unknown whether insulin acutely enhances transcription of genes or whether there is a time related pattern in transcribing the genes thereby having a different level of regulation of insulin action on gene expression. A better understanding of the impact of insulin on transcript levels of various genes is critical to acquire a more thorough understanding on how insulin exerts its pleiotropic effects on skeletal muscle glucose uptake as well as protein synthesis.

The microarray technology has been extensively used to identify differentially expressed genes in skeletal muscle cells under different physiological states, e.g., non-diabetic vs. diabetic subjects during poor glycemic control and following insulin treatment [15], insulin treated versus insulin deprived type 1 diabetic patients [16], and [15], normal *vs.* impaired glucose tolerant individuals [17], and basal state vs. euglycemic hyperinsulinemic clamp [18]. Further information on transcriptional regulation can be gained by monitoring gene transcripts related to time following insulin administration. In fact, in pre/post stimulus studies in which the transcriptional response is monitored at one specific time instant after a prolonged insulin exposure, genes showing a transient response followed by a return to the pre-stimulus expression or a systematic, but small in magnitude, change in the expression, are likely to be missed. In contrast, monitoring the dynamic response allows identifying transient responses, which might be characteristic, and, if common to a number of genes associated to the same functional group, might give insight into the function or functions performed by the gene circuitry. The aim of the present work is to exploit the potential of a dynamic study to investigate the transcriptional response of skeletal muscle cells during acute insulin stimulation. To this purpose, we designed and conducted the present gene-array experiment in differentiated L6 myotubes.

To identify significant transcriptional temporal patterns in muscle cells treated with insulin and to characterize them from a functional point of view, here we propose a new analytical method applied to experimental data. This method aims at overcoming some drawbacks of the conventional analysis approach based on selection of differentially expressed genes, clustering and functional annotation based on Gene Ontology (GO) [19]. The new approach that we apply in the current study 1) improves selection of differentially expressed genes by diminishing the number of false negatives while maintaining constant the false discovery rate, i.e. the number of false positives divided by the number of selected genes; 2) clusters genes with the same transcriptional pattern without requiring the user to fix the number of clusters and 3) automatically annotates these clusters with the most specific GO terms, avoiding redundancy of the information.

## Materials and Methods

### Skeletal Muscle Culture

L6 skeletal muscle myoblasts (purchased from ATCC, Manassas, VA) were grown and kept in low glucose (5 mM) DMEM growth media supplemented with fetal bovine serum (FBS) and an antibiotic antimycotic mixture (10% FBS, 4 mM L-Glutamine, 1.0 mM sodium pyruvate, 100 ug/ml penicillin 100 ug/ml streptomycin). Four plates were prepared for each pre-defined time sample at a density of 6∶8×10^5^cells per 100 mm dish and incubated overnight in DMEM growth media. On the second day, the DMEM growth media was changed to DMEM differentiation media with 1% FBS to initiate myotube differentiation. The differentiation media was changed every 2 days. On sixth day differentiation was completed and medium was changed to serum free DMEM for an 8 hour pre-incubation. The culture was split into 2 different groups: insulin treated and control. Just after the collection of the first biological sample at time 0′, for each time point the medium on one plate was changed to serum free DMEM with 20 nM insulin, whereas one plate was used as control (DMEM with 0 nM insulin). Cells were harvested at the designated time points by decanting off overlying medium, scraping the cells into cryovials and freezing immediately in liquid nitrogen. Samples were collected at times 0, 20, 40, 60,…, 480 minutes (every 20 minutes, for 8 hours) from both insulin treated and control cultures, for a total of 50 biological samples.

Samples (20–40–60), (80–100–120), (140,160,180), (200–220, 240), (260, 280, 300), (320,340,360) (380–400–420) and (440–460–480) from insulin-treated and control culture were pooled together, obtaining eight joint samples. Consistently, sample 0′ was harvested and collected in triplicates and pooled together (0a–0b–0c) for each culture and two targets for the hybridization were prepared separately. The pooling step was chosen to achieve a compromise between the cost of the experiment and the frequency of the sampling grid: it is safer to average the signal by pooling the biological samples than to use a sparse sampling grid (fast regulated genes would be easily lost e.g. by collecting one sample per hour).

All the culturing and sampling procedure described above was repeated two additional times on different days, using the same identical cell line, to obtain a complete triplicate of the experiment.

### Measurements

Total RNAs were purified using an RNeasy Protect Mini Kit from Qiagen. The quality and quantity of total RNA was measured using the Agilent test on a Bioanalyzer (Agilent Technologies, Palo Alto, CA). Gene transcript profiles in both control and treated cultures were studied by high-density oligonucleotide microarrays containing probes for 31,099 genes and expressed sequence tags (Rat-230.2 GeneChip arrays; Affymetrix, Santa Clara, CA), for a total of 54 chips. Sample labeling, hybridization of test array, and hybridization of full-size arrays were performed by the Mayo Clinic Advanced Genomics Technology Center Microarray Lab using protocols described in the Affymetrix GeneChip expression analysis technical manual.

The data discussed in this publication are MIAME compliant and have been deposited in NCBI's Gene Expression Omnibus [20] and are accessible through GEO Series accession number GSE28997 (http://www.ncbi.nlm.nih.gov/geo/query/acc.cgi?acc=GSE28997).

### Preprocessing

Image quantification was performed using GeneChip (Affymetrix, SantaClara, CA) scanner and software. Preprocessing steps such as background subtraction, probe cell normalization and expression level calculations, were performed using quantile normalization and Robust Microarray Analysis (RMA) software [21].

Genes were pre-filtered using Affymetrix Detection calls (genes that were not present in at least one chip were filtered out). The remaining 21958 probes were analyzed using the method described in the following for the function-based discovery of significant transcriptional temporal patterns.

### Analysis of Significant Transcriptional Temporal Patterns

A new method is here proposed to identify significant transcriptional temporal patterns, based on four different computational steps: 1) *Gene Ranking*, i.e. all genes are ranked according to a false discovery rate p-value reflecting the likelihood that the gene is differentially expressed; 2) *Functional Gene* Annotation based on GO; 3) *Search for the Temporal Patterns*, i.e. each functional group is searched for temporal patterns characterizing it; 4) *Selection of Differentially Expressed Genes*, based on both the false discovery rate (FDR) p-values and the characteristic patterns.

The output of the method is a set of clusters of differentially expressed genes, each characterized by a specific temporal pattern and by the most specific functional annotations. The four steps of the methods are described in what follows.

### Gene Ranking

Genes are ranked according to FDR p-values using a selection method of choice. In the case of the data analysis performed in this work, we used a method previously proposed [22] that calculates the area of the region bounded by the time series expression profile and assigns a p-value to the gene according to this area and a null hypothesis distribution, based on a model of the experimental error, to be derived from experimental replicates. The two replicates available at time zero were used to derive the experimental error distribution at different intensity expression values and, consequently, the null hypothesis distribution of the area bounded by the treated-minus-control expression profile.

### Functional Gene Annotation

Gene Ontology annotation is used to define the biologically relevant sets of genes. GO terms are organized in a directed acyclic graph in which each node corresponds to a GO term and may have multiple parents: nodes farther from the root correspond to more specialized terms; nodes closer to the root to less specialized terms, thus implying that genes annotated with a specific node are also annotated with every ancestor of that node.

In the case of the data analysis performed in this work, we used Molecular Function GO annotation. An additional set containing 159 genes involved in insulin signaling according to GenMapp pathway [23] was also used.

### Search for the Temporal Patterns

For each GO node, the algorithm searches the representative temporal patterns characterizing the transcriptional response. In particular, among the genes associated to a specific GO term, the algorithm searches for a subset of genes whose time series expression profile X_i_ = <x_i_(1), …, x_i_(m)> can be modeled by the following equation:
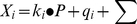
(1)where P = <p(1), …, p(m)> is the characteristic temporal expression pattern, i.e. a vector of m (number of time points) expression values, k_i_ and q_i_ are the gene i specific parameters and Σ encodes the measurement error variance. The algorithm iteratively performs a gene-specific parameter identification step and a temporal pattern search step. In the first step, the parameters k_i_ and q_i_ are identified for each gene i, using weighted least squares method. A goodness of fit test is performed for each gene i and only genes with significant p-value are kept in the cluster. In the second step, P is estimated at each sampling time, using again weighted least squares, but considering as data the k_i_ and q_i_ of the genes belonging to the cluster and estimated at the previous step. All the n genes being analyzed go again through the first step, so to identify new k_i_ and q_i_ and re-define the cluster membership based on the newly estimated pattern P. All the procedure is reiterated until the list of genes in the cluster does not change or a maximum number of iterations is reached. Each identified pattern is thus characterized by a cluster of genes with correlated profiles and the same annotation.

For each discovered pattern, the set of genes fitting this pattern (fitP) and the set of genes that do not fit it (¬fitP) are defined. Only if significant, i.e. if it contains at least one gene with false discovery rate p-value lower than a fixed threshold, e.g. 0.05, fitP is recorded as a cluster in the GO node under analysis. The procedure is then iteratively applied to ¬fitP, until ¬fitP contains no genes or no significant patterns are discovered.

Nodes are analyzed starting from the leaves of the GO graph, i.e. the nodes farthest from the root, which are the most specific GO terms; whenever a significant pattern is identified, genes correlated to the pattern are removed from all the ancestors of the node, so to avoid redundancy and annotate genes with the most specific available biological information, analogously to what has been proposed in [24]. Conversely, genes correlated to a pattern are not removed from the sibling nodes.

Further details about the method are given in (**Methods S1**).

### Selection of Differentially Expressed Genes

Given the high number of genes and the lack of replicates that usually characterize high-throughput studies, gene selection procedure has low statistical power. Moreover, the need to control the false positive rate in a multiple testing condition, e.g. by using FDR, leads to very small significant level α thus increasing the number of false negatives. To lower the percentage of false negatives, we recover, among the gene not selected as differentially expressed by a FDR threshold of 5%, those genes that: (a) have a p-value (not corrected for multiple testing) lower than 5%; are associated to a cluster of genes: i) sharing the same temporal transcriptional profile; ii) all annotated with the same functional term, iii) containing at least one gene with significant FDR corrected p-value.

2) are associated with a gene cluster containing at least 1 gene with significant FDR corrected p_value. As d above, membership to a cluster is based, beside common functional annotation, on the goodness of fit to the corresponding temporal pattern and it is statistically assessed in comparison to a flat profile.

Intuitively, since a group of genes associated to a pattern contains at least one gene with significant false discovery rate p-value, all genes in the group significantly correlated to the same temporal pattern, significantly different from a flat profile and sharing the same functional annotation are likely to be differentially expressed.

### Simulated Data

**Figure 1:**
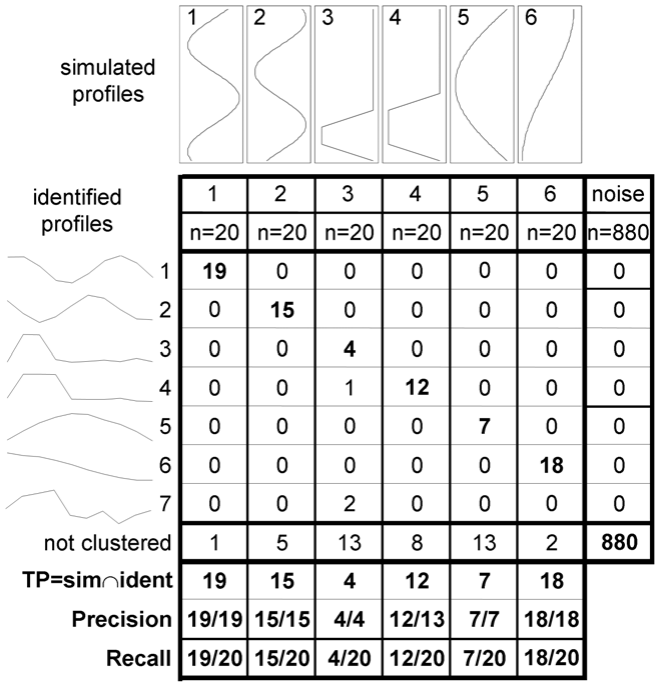
Temporal patterns used to simulate the data (columns) and identified by the method (rows) for a specific simulated data set. 100 data sets of 1000 genes monitored on 13 time samples were simulated. Each data set, like the one shown in this figure, consists of 120 differentially expressed genes separated in 6 clusters characterized by different temporal patterns and 880 not differentially expressed profiles. The number of genes in the intersection between a simulated and an identified cluster is indicated; the maximum of these numbers for each simulated cluster is assumed as the number of true positives. For each column, precision is calculated as true positives divided by the number of genes identified in the cluster; recall as true positives divided by the number of genes in the simulated cluster.

100 data sets of 1000 genes monitored on 13 time samples were simulated. Each data set consists of 120 differentially expressed genes separated in 6 clusters characterized by different temporal patterns (**Figure 1**) and 880 not differentially expressed profiles. Patterns 1, 2 and 3 in **Figure 1**, vary between −1 and 1; whereas patterns 4, 5 and 6 vary between 0 and 1. Differentially expressed gene profiles were generated from the pre-defined patterns, using the model described in Eq. (1): for each pattern 20 gene profiles were generated: 10 with k_i_ and q_i_ sampled from uniform distributions in the intervals ±(0.5, 2) and (−0.5, 0.5), respectively, and 10 with k_i_ and q_i_ sampled from uniform distribution in the intervals ±(1, 3) and (−3, 3), respectively. Gaussian noise was added to all data with mean 0 and standard deviation equal to 0.2 (the range of expression of genes varies at maximum between −6 and 6).

## Results

### Method validation

The ability of the method to identify groups of genes belonging to the same pattern was assessed on synthetic data by comparing identified to simulated clusters, in terms of precision (number of correctly classified genes among the inferred ones) and recall (number of correctly classified genes among the true ones). **Figure 1** illustrates the characteristic patterns used to generate the data (columns) and the results (rows) for one representative data set among the 1000 simulated, containing 6 clusters of 20 genes each, and 880 noisy expression profiles. The number of genes in the intersection between a simulated and an identified cluster is indicated; the maximum of these number for each simulated cluster is assumed as the number of true positives, that is the measure of the ability to identify clusters with all or most genes belonging to a single (simulated) cluster. For each column, precision is calculated as true positives divided by the number of genes identified in the cluster; recall as true positives divided by the number of genes in the simulated cluster. The average results on the 100 simulated datasets are shown in **Table 1**.

**Table 1:** Results of the clustering on simulated data.

Clusters:	1	2	3	4	5	6
Precision	98%	99%	91%	96%	97%	98%
	(2%)	(1%)	(9%)	(3%)	(1%)	(1%)
Recall	85%	86%	33%	49%	35%	85%
	(8%)	(8%)	(14%)	(11%)	(13%)	(7%)

Average precision and recall results on 100 simulated data sets; standard deviation in parenthesis.

Since the method was applied in a pipeline with a selection procedure, it is also of interest to assess its ability to select differentially expressed genes. In average, on 100 simulations the number of false negatives diminishes from 11% to 9% (p-value = 0.0091, Wilcoxon test) in correspondence of a constant false discovery rate (number of false positives divided by the number of selected genes) of 5%. Therefore, the pattern search applied in combination with the selection method, besides being able to identify the main temporal patterns of expression, improves the selection by lowering the percentage of the false negatives.

### Transcriptional Temporal Patterns in Insulin Stimulated Muscle Cells

**Figure 2:**
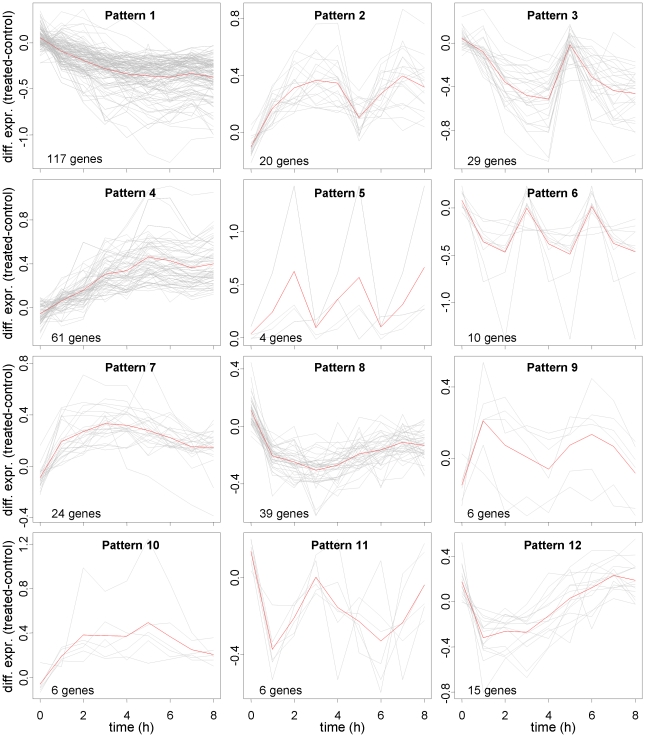
Expression profile of genes selected as differentially expressed clustered in groups of genes sharing the same temporal patterns. The average differential expression profiles (treated minus control) of the genes in the different clusters is shown in red; the number of genes in each cluster and their differential expression profile (in gray) is also reported for each cluster.

**Figure 3:**
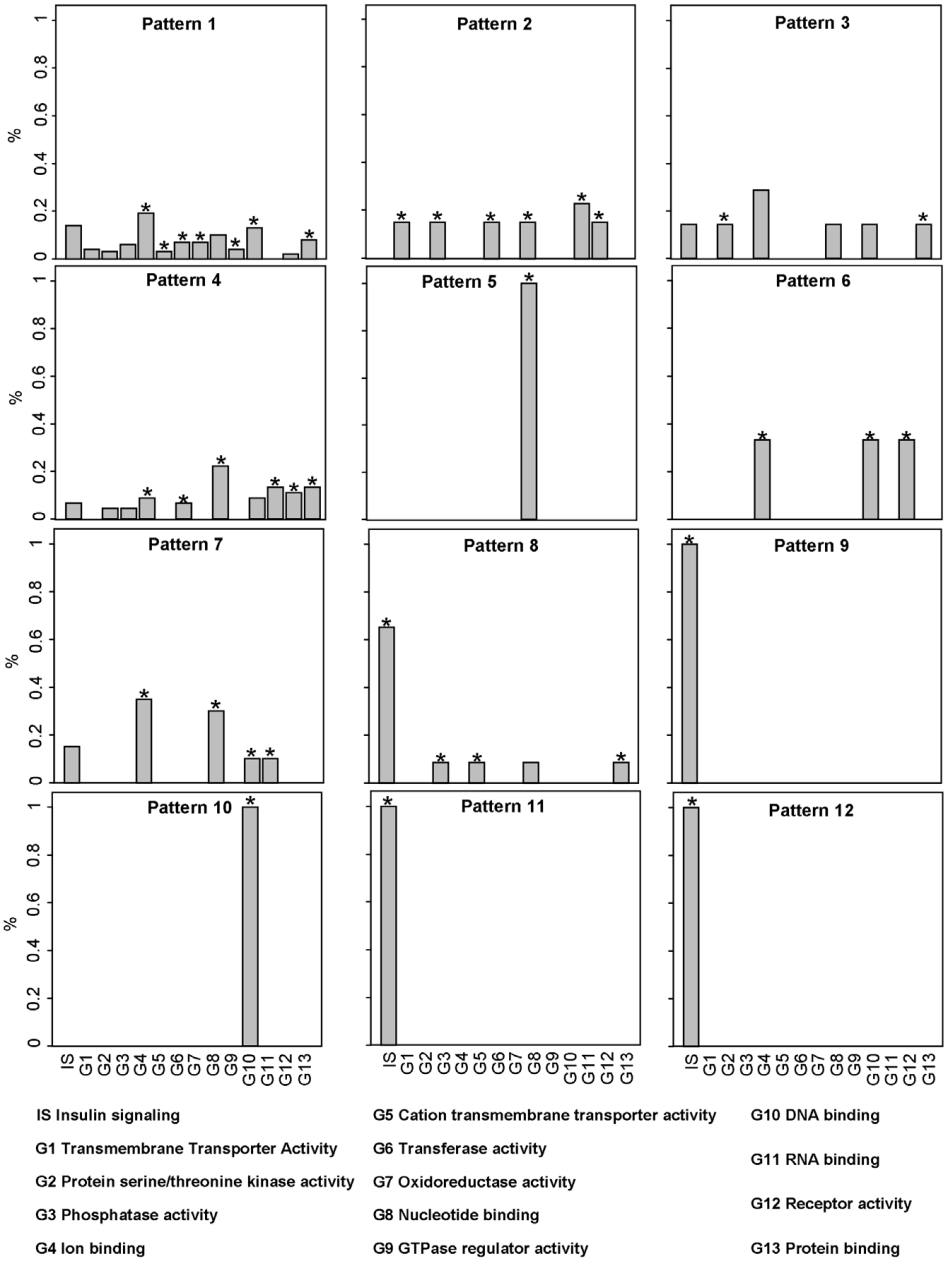
Percentage of genes belonging to insulin signaling pathway (IS) and to 13 different GO groups. Each group is defined as a set of nodes directly connected in the GO graph and labeled with the most general of these nodes. Enriched GO terms (p-value<0.05) are highlighted with a star symbol.

326 genes were selected as differentially expressed and clustered into 12 different clusters, each characterized by a specific expression pattern. **Figure 2** shows in red the average differential (treated minus control) expression profiles of the genes in the different clusters; the number of genes in each cluster and their differential expression profile (in gray) is also reported. A detailed list of selected genes, their annotation with the GO molecular function term and the associated patterns is available in **Table S1** (**Tables S1.1, S1.2, s1.3, S1.4, S1.5, S1.6, S1.7, S1.8, S1.9, S1.10, S1.11, S1.12, S1.13**). To obtain a more synthetic annotation, the GO nodes directly connected by a path in the GO graph were grouped, thus obtaining 13 GO groups plus the group of genes annotated to insulin signaling. Each GO group, thus characterized by an isolated sub-graph of siblings or ancestors terms, was labeled with the most general of these terms. Genes can appear annotated in more than one GO term and/or in more patterns since, as explained in Method, a gene associated to a pattern and a GO term is deleted from the ancestor of the GO node it belongs to, but not from its siblings. **Figure 3** shows, for each pattern, the percentage of genes belonging to insulin signaling pathway (IS) and to each GO group (G1–G13). GO enrichment analysis based on Fisher's Exact Test was also performed to highlight the most relevant GO terms associated with the genes belonging to each pattern with respect to the total number of selected genes. Enriched GO terms (p-value<0.05) are highlighted with a star symbol in **Figure 3**.

104 genes annotated to GO nodes isolated in the GO tree, i.e. without sibling or parent nodes found as significant after the analysis, are not shown in **Figure 3** and in **Tables S1.1, S1.2, S1.3, S1.4, S1.5, S1.6, S1.7, S1.8, S1.9, S1.10, S1.11, S1.12, S1.13**; complete results and information on selected genes, gene ontology terms and differential expression data associated to each pattern are available in **Table S2**.

Genes belonging to patterns 1 and 4, which are also the most numerous, are annotated to many different GO groups, of which many are also significantly enriched. Patterns 5, 9, 10, 11 and 12, on the opposite, are characterized by genes belonging to a single GO group: nucleotide binding (G8) for pattern 5, RNA binding (G11) for pattern 10 and insulin signaling (IS) for patterns 9, 11 and 12. Patterns 2, 3, 6, 7 and 8 are in an intermediate situation, with genes annotated to a number of GO groups ranging from 3 to 6.

Looking at the GO groups associated to a limited number of patterns, we found that transmembrane transporter activity (G1) is characterized by 2 genes belonging to pattern 1 and 2 to pattern 2; protein serine/threonine kinase activity (G2) by 3 genes belonging to pattern 1, 2 to pattern 4 and 2 to pattern 3, these latter with kinase inhibitor activity; Cation transmembrane transporter activity (G5) is characterized by 3 genes belonging to pattern 1, and 2 to pattern 8. Oxidoreductase activity (G7) and GTPase activity (G9) contains genes associated only to pattern 1.

**Figure 4:**
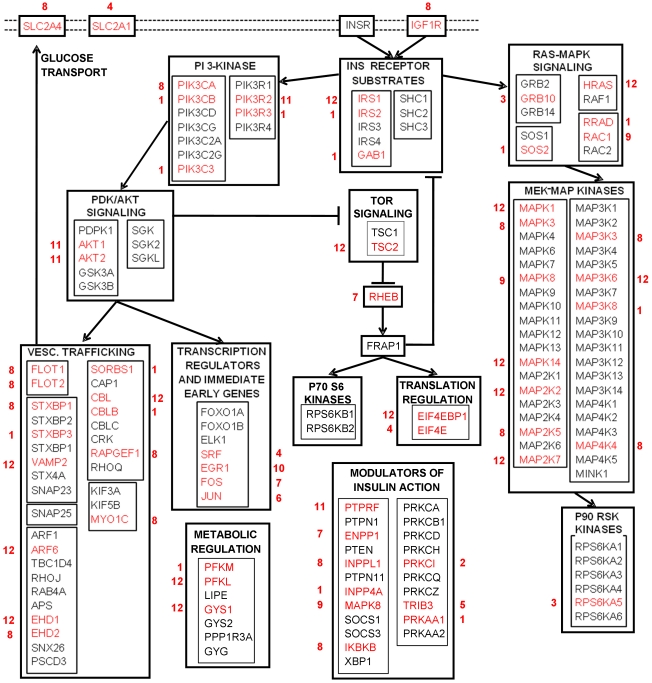
Mapping of differentially expressed genes on the insulin signaling pathway. 62 genes (in red) belonging to the insulin signaling pathway (GenMapp), selected as differentially expressed and associated with significant temporal expression patterns.

Interestingly, 62 of the 326 selected genes belong to insulin signaling pathway, defined according to GenMapp annotation. They are shown in **Figure 4** in red, together with the identifier of their cluster. Most of them are associated with pathways showing down-regulation in treated vs. control cultures such as pattern 1, 8 and 12. In details, 14 genes are associated with pattern 1, 1 with pattern 2, 2 with pattern 3, 3 with pattern 4, 1 with pattern 5, 1 with pattern 6, 3 with pattern 7, 15 with pattern 8, 2 with pattern 9, 1 with pattern 10, 4 with pattern 11 and 15 with pattern 12. Among these genes, glycogen synthase 1 (GYS1) had not a significant false discovery rate p-value, but it was selected because highly correlated with other 14 significant genes in cluster 12, all belonging to insulin signaling node.

## Discussion

In the current study we applied a gene array profiling approach to the transcriptional response of skeletal muscle myotubes treated with insulin, to identify the main temporal expression patterns associated to functional groups of differentially expressed genes. We chose skeletal muscle cell line for this study considering that frequent sampling from human and animal studies are fraught with many logistical problems including ethical issues. The results from the current study knowing the critical time points at which the transcriptional responses are altered by insulin will enable planning of future *in vivo* studies to assess insulin's effect on muscle gene transcripts. A prior time course of insulin response in primary human myotubes from type 2 diabetic and non-diabetic individuals (0, 0.5, 1, 2, 4, 8, and 24 hours) by Hansen et al. [25] demonstrated a time dependent transcriptional responses of inflammatory and pro-angiogenic pathways in relationship to glycogen synthesis. The authors identified 102 transcripts as differentially expressed in response to insulin some of which were similar to those among the 326 gene transcripts that were differentially expressed in our analysis. For example, the angiogenic/anti-apoptotic gene transcripts, VEGF, FOS, and SRF, were up-regulated in both studies. Comparison of the above study to the current study is difficult since different species and cell types were used, different insulin concentrations (20 nM vs. 1 µM) were used, and the statistical analysis and the objectives of the two studies differ. The analysis in the current study aimed at integrating selection, clustering and functional annotation to find the main temporal patterns associated to functional groups of differentially expressed genes.

Our method was applied using a previously proposed method for gene selection [22], but different choices are possible, as long as the selection method provides a list of p-values associated to the list of analyzed genes. Analogously, Gene Ontology functional annotation or different annotation databases can be used. If the functional annotation is hierarchical, it has to be codified in a hierarchical direct graph as in the case of GO, before the search of temporal patterns is performed. To associate patterns to the most specific Gene Ontology nodes and avoid redundancy of the information, our method starts searching patterns from the leaves of the GO graph (i.e. the nodes farthest from the root, which are the most specific) and whenever a significant pattern is identified, genes correlated to the pattern are removed from all the ancestors of the node. The pattern search is based on a linear model whose parameters are identified using least squares, thus it accounts for the error measurement, does not require the user to fix the number of clusters and is not computationally demanding. Our method improves the selection procedure by combining information on false discovery rate p-value with both functional group and characteristic temporal pattern association, thus diminishing the number of false negatives without significantly increasing the number of false positives. Previous works have addressed the integration of prior knowledge either in clustering algorithms [26]–[28] or in selection procedures [29], whereas our approach integrates selection, clustering and annotation in a single computational framework.

Based on our analysis, 326 genes were selected as differentially expressed and 12 different patterns of gene expression profile were identified. For example, the expression pattern 1 (**Figure 2**) shows a slow and gradual decrease in gene expression whereas in pattern 4 there is a gradual increase in gene expression reaching a peak at about 5 hours and then reaching a plateau. These patterns are annotated with a number of functional annotation (**Figure 3**). In particular, pattern 1 is enriched with GO groups Ion binding, Cation transmembrane transporter activity, Transferase activity, Oxidoriductase activity, Nucleotide binding; GTPase regulator activity, DNA binding and Protein binding; whereas pattern 4 is enriched with GO groups Ion binding, Transferase activity, Nucleotide binding, RNA binding, Receptor activity and Protein binding. Interestingly pattern 3, showing an initial decrease in gene expression followed by a variable pattern of increase in gene expression over time, is enriched with GO group Phosphatase activity and is anticorrelated with pattern 2, enriched with GO group Protein serine/threonine kinase activity. Besides generic annotation as Protein binding or RNA binding, pattern 2 is also enriched with GO groups Transmembrane transporter activity, Transferase activity and Receptor activity. All genes in pattern 5 are annotated as Nucleotide binding, all genes in pattern 10 as DNA binding. More interestingly, all genes in patterns 9, 11 and 12 are annotated in Insulin signaling pathway, with patterns 9 and 11 anticorrelated. 65% of the genes in pattern 8 are also annotated in Insulin signaling pathway; the remaining genes associated to pattern 8 are annotated in GO groups Phosphatase activity and Cation transmembrane transporter activity. Finally, patterns 6 and 7 are enriched with GO group Ion binding; pattern 6 also with GO group Receptor activity. The identified patterns are followed rather tightly by the genes in each cluster. Such a consistent behavior supports our confidence on the reliability of the cluster average pattern. Moreover the three replicated experiments show high data reproducibility: the variance calculated across replicates for the 326 genes selected as differentially expressed has median value equal to 7% with first and third quartiles equal to 0.03 and 0.15 respectively.

Approximately 20% of the genes that were differentially expressed were identified as belonging to the insulin signaling pathway (**Figure 4**). Of interest, most of the genes were down-regulated in response to insulin treatment under the present experimental conditions. The results that demonstrate that IRS-1 mRNA abundance is initially down-regulated (pattern 12) with insulin treatment is consistent with the recent observation the IRS-1 mRNA abundance was down-regulated following a three hour insulin infusion during an *in vivo* hyperinsulinemic-euglycemic clamp [30]. It should be noted that after 6 h of treatment the IRS-1 mRNA abundance returned to baseline, followed by a slight increase in mRNA abundance above baseline between 6 and 8 h. However, the present results are in contrast to the recent finding that genes involved in insulin signaling were largely up-regulated in response to a three hour insulin infusion during an *in vivo* hyperinsulinemic-euglycemic clamp [18]. Moreover, our results that IRS-2 mRNA abundance is down-regulated (pattern 2) in response to insulin treatment *in vitro* under the present experimental conditions is in contrast to modest increase in IRS-2 mRNA abundance in response to a four hour insulin infusion during an *in vivo* hyperinsulinemic-euglycemic clamp [31]. In addition, the angiogenic/anti-apoptotic gene transcripts, VEGF, FOS, and SRF, were up-regulated in response to the insulin treatment, which is consistent with the findings of Hansen and colleagues [25]. Greenhaff and colleagues have recently reported [32] AKT (PKB) mRNA abundance remains unchanged following three hours of hyperinsulinemia under four different steady-state insulin concentrations range from 5 mU/l to ∼170 mU/L. The mRNA (transcripts) expressions in the current study and other studies represent the net changes related to production and degradation of mRNA. It is possible that insulin's primary effect is translation of the transcripts involved in glucose metabolism. A higher rate of transcription than translation of these genes would have resulted in higher transcript levels. In addition, insulin also stimulates skeletal muscle glucose uptake by the phosphorylation of specific signaling proteins involved in glucose metabolism in skeletal muscle [33].

The present results also indicate that insulin treatment *in vitro* stimulates gene expression changes that likely promote protein synthesis. Specifically, insulin treatment resulted in down-regulation of mRNA abundance of TSC2 (pattern 12), which is a known negative regulator of protein synthesis. Mechanistically, TSC1 (hamartin) forms a complex with TSC2 (tuberin) [TSC1–TSC2 complex], which functions as a critical regulator of protein synthesis and cell growth [34], [35]. Indeed, loss-of-function mutations in TSC2 have been shown to reduce mTOR and s6k activity [36]. Moreover, insulin treatment resulted in up-regulation of mRNA abundance of Rheb (pattern 7), which is known positive regulator of protein synthesis [37]. Mechanistically, Rheb-GTP binds directly to the mTOR kinase domain, which in turn activates mTOR's catalytic function [30]. Insulin treatment also likely promotes translational initiation by down-regulating mRNA abundance of EIFBP1 (pattern 12), while simultaneously up-regulating mRNA abundance of EIF4E (pattern 4). These findings are consistent with recent finding reported by Colleta and colleagues [31] who observed an increased mRNA abundance for EIF4E following a four hour insulin infusion during an *in vivo* hyperinsulinemic-euglycemic clamp. Furthermore, insulin treatment resulted in up-regulation of mRNA abundance of DNA-directed RNA polymerase I polypeptides E (pattern 4), B (pattern 4), C (pattern 7) and DNA-directed RNA polymerase II polypeptide C (pattern 7) [**Table S1.10**] as well as mRNA abundance of several RNA binding proteins (patterns 2, 4 and 7) [**Table S1.11**].

The current *in vitro* experiments cannot be directly translated in to *in vivo* situation in either human or animals. First, the present experiments were conducted in differentiated L6 myotubes and not in fully developed skeletal muscle fibers. Second, the differentiated L6 myotubes were incubated in normal-glucose (5 mM glucose) in the presence of relatively high insulin concentrations (20 nM) in order to elicit maximal effect in an *ex-vivo* study. Moreover, the present experiments were conducted in a serum starved state. Therefore, the changes in mRNA transcripts both in the treated and control conditions likely also reflect the effects of progressive serum starvation. The responses may be different in hyperglycemic conditions with lower or higher insulin concentrations. Moreover, insulin is known to reduce protein degradation and amino acid levels [38] Therefore, future *in vivo* studies are warranted that examine the effect of hyperinsulinemia while maintaining both euglycemia and euaminoacidemia.

The present investigation demonstrates a gene array experimental design and methodology for examining temporal changes in gene expression. Using gene array profiling, we identified 12 different temporal patterns of gene expression in response to eight hours of insulin treatment *in vitro*. These results are likely to help design of *in vivo* studies to examine the effect of insulin treatment on the temporal regulation of gene expression related to glucose uptake in human and animals. Finally, the insulin treatment affected not only the transcripts involved in glucose metabolism but also stimulated gene transcripts that would promote protein synthesis.

## Supporting Information

Methods S1 Detailed methods on the search for temporal patterns, which includes a detailed description of the search algorithm.
(DOC)

Table S1 A detailed list of selected genes, their annotation with the GO molecular function term and the associated patterns.
(DOC)

Table S2 A complete list of annotated genes along with the information on the selected genes, gene ontology terms and differential expression data associated to each pattern.
(XLS)
