# Supplementary material for: Function-Based Discovery of Significant Transcriptional Temporal Patterns in Insulin Stimulated Muscle Cells
Source: PLoS One. 2012 Mar 1;7(3):e32391. doi: 10.1371/journal.pone.0032391 (PMC3291562; doi:10.1371/journal.pone.0032391)
Supplement: Methods S1 — Detailed methods on the search for temporal patterns, which includes a detailed description of the search algorithm. (DOC) [file pone.0032391.s001.doc]

***Search for the Temporal Patterns* – *Algorithm Details***

For each node in the GO graph, the main temporal patterns characterizing the genes annotated in the node are searched. Each identified pattern is characterized by a cluster of genes with correlated profiles and the same annotation.

Nodes are analyzed starting from the leaves of the graph, i.e. the nodes farthest from the root, which are the most specific GO terms; whenever a significant pattern is identified, genes correlated to the pattern are removed from all the ancestors of the node, so to avoid redundancy and annotate genes with the most specific available biological information, analogously to what has been proposed in (Alexa et al., 2007). Conversely, genes correlated to a pattern are not removed from the sibling nodes.

The search for patterns in each node is performed using two different computational modules: the first, called “Find All Patterns” (FAP), to cluster genes sharing the same expression pattern and functional annotation through the GO graph (we call these clusters SAP, i.e. “Share Annotation and Pattern” in the following); the second, called “Find the Main Pattern” (FMP), to identify the most significant characteristic pattern in a group of genes.

FAP (**Figure S1.1**) takes as input: 1) the expression data, i.e. an n×m matrix where each row contains the time series expression profiles Xi=<xi(1), ..., xi(m)> of a gene i (i=1, ..., n); 2) the list of the n monitored genes ranked according to false discovery rate p-value; 3) the n×m matrix E containing the information on the error variance; 4) the gene annotation. It iteratively searches the main characteristic patterns in each group of genes associated to the GO terms given as input. For each discovered pattern, the set of genes fitting this pattern (fitP) and the set of genes that do not fit it (ØfitP) are defined (**Figure S1.1**). Only if significant - i.e. if it contains at least one gene with false discovery rate p-value lower than a fixed threshold, e.g. 0.05 - fitP is recorded as a SAP cluster in the GO node under analysis. The procedure is then iteratively applied to ØfitP, until ØfitP contains no genes or no significant patterns are discovered. The genes in SAP are deleted from the GO term being analyzed and from all its ancestors.

**Figure 4. Pseudocode description of the algorithm “Find All Patterns”** (**FAP).** FAP iteratively searches the main characteristic patterns in each group of genes associated to the GO terms given as input. For each discovered pattern, the set of genes fitting this pattern (fitP) and the set of genes that do not fit it (¬fitP) are defined using the function “Find the Main Pattern” (FMP). If fitP contains at least one gene with false discovery rate p-value lower than a fixed threshold, fitP is recorded as a SAP cluster in the GO node under analysis. The procedure is then iteratively applied to ¬fitP, until ¬fitP contains no genes or no significant patterns are discovered. The genes in SAP are deleted from the GO term being analyzed and from all its ancestors.

The core of the method is the search for the most significant characteristic pattern in a set of genes, implemented in the function FMP. This function requires as input a set of gene expression profiles and the error matrix E. It searches for a subset of genes whose time series expression profile Xi=<xi(1), ..., xi(m)> can be modeled by the following equation:

$$X_{i}=k_{i}\cdot P+q_{i}+\Sigma$$

where P=<p(1), ..., p(m)> is the characteristic time pattern, ki and qi are the gene specific parameters of the model and Σ

is the covariance matrix of the error. The cluster and the temporal pattern P=<p(1), ..., p(m)> are initialized with the time series expression profile of the first gene given as input, i.e. the one with the most specific p-value reflecting the likelihood it is differentially expressed (during the first iteration P is updated as the average time series expression profile across all genes joining the cluster).

The function FMP iteratively performs a gene-specific parameter identification step (M step) and a temporal pattern search (E step). In the M step, the parameters k_i_ and q_i_ are identified for each gene i, using weighted least squares method. A goodness of fit test is performed for each gene i and only genes with significant p-value are kept in the cluster. In the E step, P is estimated at each sampling time, using again weighted least squares, but considering as data the k_i_ and q_i_ of the genes belonging to the cluster and estimated at the previous step. All the n genes being analyzed go again through the M step, so to identify new k_i_ and q_i_ and re-define the cluster membership based on the newly estimated pattern P. All the procedure is reiterated until the list of genes in the cluster does not change or a maximum number of iterations is reached.

In the case of the data analysis performed in this work, we assume the error independent for different genes and different time samples, mainly because replicates of the entire experiment were not available in sufficient number to derive a gene specific estimate of the error variance. The covariance error matrix Σ is therefore diagonal with elements given by E(i,•). The nature of the matrix E depends on the available noise knowledge and ultimately, on the available experimental replicates. Frequently in microarray studies, a dependency of the noise level from the intensity of gene expression is observed (Tu et al., 2002). This information can also be codified in the n×m matrix E, by expressing the variance of each gene i (i=1,... , n) at time sample t (t=1,..., m), as dependent on its intensity value xi(t).

The algorithm described above searches for characteristic patterns in subsets of genes pre-defined on the basis of their functional annotation. However, this strategy could lead to represent the same temporal pattern with two slightly different profiles in a parent and a child node or in two sibling nodes, just because different genes were used to discover the pattern in the two nodes. To overcome this limitation, when the last GO node (the root) has been analyzed, all the discovered SAP patterns are analyzed using the procedure FAP, described above and the patterns are re-defined based on the new discovered characteristic temporal profiles, without however modifying the set of genes in each SAP.

- Alexa A, Rahnenführer J, Lengauer T (2006) Improved scoring of functional groups from gene expression data by decorrelating GO graph structure. Bioinformatics 22: 1600–1607.
- Tu Y, Stolovitzky G, Klein U (2002) Quantitative Noise Analysis for gene expression microarray experiment. Proc Natl Acad Sci 99: 14031-14036.
