# Supplementary material for: Function-Based Discovery of Significant Transcriptional Temporal Patterns in Insulin Stimulated Muscle Cells
Source: PLoS One. 2012 Mar 1;7(3):e32391. doi: 10.1371/journal.pone.0032391 (PMC3291562; doi:10.1371/journal.pone.0032391)
Supplement: Table S1 — A detailed list of selected genes, their annotation with the GO molecular function term and the associated patterns. (DOC) [file pone.0032391.s002.doc]

**Table S1.1. Transmembrane Transporter Activity**

**Table S1.2. Protein serine/threonine kinase activity**

**Table S1.3. Phosphatase activity**

**Table S1.4. Ion binding**

**Table S1.5. Cation transmembrane transporter activity**

**Table S1.6. Transferase activity**

**Table S1.7. Oxidoreductase activity**

**Table S1.8. Nucleotide binding**

**Table S1.9. GTPase regulator activity**

**Table S1.10. DNA binding**

**Table S1.11. RNA binding**

**Table S1.12. Receptor activity**

**Table S1.13. Protein binding**
